# “It’s probably like a web”: complex relationships between pain and mental health among bisexual+ women with chronic pain

**DOI:** 10.3389/fpsyt.2026.1811843

**Published:** 2026-07-29

**Authors:** Aster Harrison, Mansha Mirza

**Affiliations:** 1 College of Applied Health Sciences, Department of Disability and Human Development, Department of Occupational Therapy, University of Illinois at Chicago, Chicago, IL, United States; 2 College of Rehabilitation Sciences, Department of Occupational Therapy, Thomas Jefferson University, Philadelphia, PA, United States; 3 Faculté des Sciences Médicales et Paramédicales, Centre d’études et de recherche sur les services de santé et la qualité de vie (CEReSS), Aix-Marseille Université, Marseille, France

**Keywords:** bisexual health, chronic pain, disability studies, LGBTQ health, mad studies, mental health, social determinants of health, women’s health

## Abstract

**Introduction:**

Bisexual+ women experience higher rates of both mental illness and chronic pain when compared to either straight or lesbian women. Their experiences of the relationship between their mental health and chronic pain remain underexplored. This article presents an intersectional Interpretive Phenomenological Analysis of the experiences of six bisexual+ women with chronic pain.

**Methods:**

Each woman completed two semi-structured interviews. Interviews were analyzed with Interpretive Phenomenological Analysis, beginning with in-depth case study analysis of each person’s interviews before moving to comparison across cases.

**Results:**

Results indicated a complex web of multidirectional relationships between mental health and chronic pain. These relationships were fraught because of the cultural context which often dismisses women’s pain as “in their heads”. Pain could worsen mental health but also offer insights. Mental unwellness could worsen participants’ ability to care for their pain. Sociocultural stressors, trauma, and relationship stress were described as worsening both mental health and chronic pain. Emotional release could sometimes help with pain relief.

**Discussion:**

Results are discussed in the context of minority stress and embodiment frameworks. Chronic pain can be a manifestation of minority stress in the bodies of bisexual+ women with chronic pain, and bisexual+ women themselves feel and identify these links between sociocultural stressors and pain. Participants highlight the importance of believing bisexual+ women with chronic pain, as well as the need for both holistic medical care and social change.

## 1 Introduction

Bisexual+^1^ women experience higher rates of intimate partner violence, sexual assault, substance use, and several mental and physical health conditions as compared to heterosexual and/or lesbian peers (1). Recent research suggests disproportionately high rates of chronic pain among bisexual+ people as compared to heterosexual and gay/lesbian people (2–6). Data from a representative sample of US adults found that individuals who identify as bisexual or “something” else have significantly higher general pain prevalence than both gay/lesbian or heterosexual adults. In addition, rates of pain in three or more body parts were twice as high among bisexual people as compared to heterosexuals (6). Another analysis of nationally representative data reported age-adjusted prevalence of chronic pain to be 32.9% among bisexual adults, compared to 20.7% of gay/lesbian adults and 19.3% among heterosexuals (3).

Mental health disparities among bisexuals, particularly bisexual women, are well-established in the literature, including higher rates of depression, anxiety, suicidality, substance use disorders, and post-traumatic stress disorder (PTSD) (1, 7, 8). Emerging literature also suggests that nonbinary and transgender bisexual individuals are at particularly high risk for mental health disparities (9, 10). Research with populations that are not exclusively bisexual has established frequent comorbidity between mental illness and chronic pain diagnoses (11–13). Links also exist between experiences of trauma and PTSD and chronic pain (14). This is particularly relevant to bisexual people, who experience higher rates of sexual violence and intimate partner violence (IPV) (15). People who have experienced IPV including rape, physical violence, or stalking are more likely to experience headaches and chronic pain than counterparts who have not experienced such IPV (16).

Pain in bisexual individuals can be linked to biphobic discrimination^2^, especially among transgender people (17). Biphobic and sexist discrimination is also a known contributor to negative mental health outcomes for bisexual women (1). Disproportionate rates of certain health problems among bisexual women have been understood by scholars using minority stress models (1, 18), which explain that stigma and discrimination serve as daily stressors which increase physiological and mental stress responses, and thereby risk for negative health outcomes (19, 20). While quantitative data about bisexual mental health disparities has grown in the past decade, and data about chronic pain disparities has emerged in the last few years, less is known about bisexual+ women’s qualitative experiences of living with chronic pain, including their perceptions of the relationships between chronic pain and mental health. This qualitative study examined bisexual+ women’s experiences of chronic pain using an intersectional approach informed by Disability Studies.

## 2 Methods

This methods section is organized in accordance with the COREQ criteria (21).

### 2.1 Methodological framework

This study used an Interpretive Phenomenological Analysis (IPA) approach. IPA requires rich analysis of each participant as a specific case study prior to making comparisons across cases (22). This unique attention to each participant and to differences between participants allows the researcher to avoid generalizations of minority experiences.

### 2.2 Participant selection

Participants were recruited via email lists and Facebook groups for local bisexual organizations, with permission from organizers. Participants were eligible if they identified as “bisexual+”, identified as a woman^3^, and had persistent or frequent chronic pain lasting at least six months. Over sixty people responded to the recruitment posts and completed a screening survey, from which six bisexual+ women with chronic pain were purposively sampled to include both transgender and cisgender women from different age brackets and racial/ethnic backgrounds. To allow for thorough analysis of each person’s case, small samples of three to six participants are recommended in IPA (22).

### 2.3 Setting and data collection

Two semi-structured interviews lasting from 75 to 150 minutes were completed with each participant. A demographic questionnaire and two separate interview guides for the initial and follow-up interviews were developed by the first author and later piloted with one bisexual+ woman with chronic pain. Interview questions and prompts were refined based on feedback from the pilot participant and the first author’s dissertation committee comprising experts in qualitative interviewing. The pilot testing of the interview guide was not audio recorded, and pilot tester responses were not used as data. A separate member checking guide was also developed based upon review of the literature and consideration of feedback from the dissertation committee and pilot tester. These materials are all available as appendices elsewhere (23).

All interviews took place in the same large city in the United States. Initial interviews occurred in person in Fall and Winter of 2019. Interviews took place in a private location of the participants’ choosing - either on a university campus, at a public library, or at the participant’s home. Follow-up interviews with two participants were completed in person in early 2020. Follow-up interviews with remaining participants were completed by video conference in Spring 2020 due to the onset of the COVID-19 pandemic. All interviews were audio recorded and transcribed. Otter AI transcription software (24) was used to make an initial draft transcription for first and second interviews. Then, a human researcher manually checked each transcript, listening to audio and correcting transcription with a transcription pedal. Verbatim transcriptions were made, maintaining hesitations, filler words, and repetitions. The first author wrote field notes and reflexive journal entries throughout data collection and analysis.

### 2.4 Data analysis

**Figure 1:**
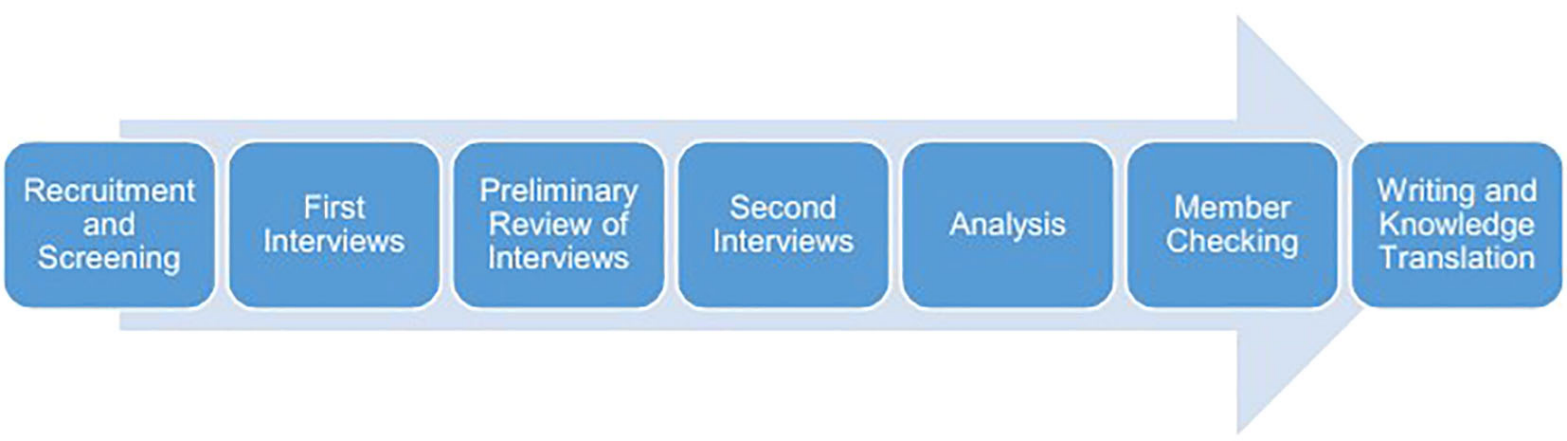
Research design. An image of an arrow points from left to right to indicate the progress through steps of the research. The steps are sequentially aligned as follows: recruitment, first interviews, preliminary review of interviews, second interviews, analysis, member checking, writing and knowledge translation Reproduced by Harrison, 2022 (23).

The research design is shown in **Figure 1**. After recruitment, screening, and first interviews, researchers conducted a preliminary review of interviews. The first author relistened to interview audio while making descriptive and interpretive notes. They identified areas where more depth was needed, or important topics discussed by some participants and not others and constructed second interview guides accordingly. Then, full data analysis was completed after the second interviews. Analysis followed the hermeneutic approach of IPA, with attention paid both to differences and similarities across interview transcripts. Analysis was completed by the first author with feedback on themes provided by the second author. Analysis followed the methodological guidance of Smith, Flowers and Larkin in the 2009 IPA manual (22), as the updated 2022 version (25) had not yet been published. The process nonetheless aligns well with current approaches, in which individual case analysis of each participant is completed first, moving from exploratory notes to experiential statements (previously called “emergent themes” in the 2009 edition) to personal experiential themes (previously “superordinate themes”). Only after each individual’s interviews had been analyzed did the analysis move to comparison across participants, flowing toward the identification of group experiential themes (25, 26).

The first author began analysis by rereading transcripts and relistening to interview audio while making exploratory notes. Based on these exploratory notes, a short summary of each interview was written (27). Interview transcripts were then imported into Atlas.ti 8 Qualitative Software (28) and coded twice. Codes represented experiential statements and organized related experiential statements into personal experiential themes. After coding both interviews for each participant, the process started fresh (not reusing exploratory notes, experiential statements, or personal experiential themes) for the next participant. After individual analysis was completed for all participants, cross-case analysis began. First, all interview summaries were re-read. Then, experiential statements and personal experiential themes from individual cases were organized into group experiential themes. Group experiential themes were designed to represent individual complexities and contradictions from multiple participants on a given topic.

Final themes were organized into PowerPoint presentations with exemplifying anonymized quotations to share with participants at credibility checking/member checking sessions. Five participants completed member checking sessions where they reviewed the group experiential themes and offered feedback. While member checking is not a standard part of IPA, these sessions allowed the research team to address participant safety and ensure the results were credible. All member checking sessions were completed by video conference in the Summer of 2021. No participants disagreed with major themes. Some participants chose to change a pseudonym or mask certain details in their quotes for increased anonymization before publication. Participants offered feedback on the importance of the results and their ideas for dissemination. Participants expressed discomfort with the presentation of quotations with repetitions and filler words, worrying that these natural imperfections in their speech rendered the quotations less readable and would lead to their testimony being taken less seriously. In response to this feedback, the first author edited final published quotations to remove hesitations and filler words that were not significant to the interpretation of the quotation. This editing process is described in greater detail elsewhere (23). Member checking audio was recorded and subsequently transcribed with Otter AI. Unclear portions of the transcripts and any quotations used for final publications were manually checked by the first author.

The multiple interviews and member/credibility checks allowed for greater depth of engagement with each participant and served as a feminist disability studies approach to co-construction of research knowledge. IPA describes a “double hermeneutic”, in which the researcher makes sense of the participants’ own sense-making (22, 25, 26). The multiple interviews allowed participants an additional opportunity to participate in the sense-making. For example, sometimes when asked about a topic that had been discussed with other participants, participants readily offered interpretations of the similarities and differences between participant experiences. Some of these examples of co-construction are highlighted in the results that follow. Given the historical and present experiences of oppression of bisexual people, women, and people with chronic pain, additional back-and-forth engagement with the participants promoted trust, safety, and shared ownership of findings.

Saturation was not considered for this research, as it is not generally used in IPA research. Methods to ensure rigor included negative case analysis, reflexivity, supervision, and member checking.

### 2.5 Research team and reflexivity

All interviews and member checking sessions were completed by the first author, who at the time had clinical master’s and doctoral degrees in occupational therapy and several years of training in qualitative research through work as a research assistant and graduate student, primarily in the research lab of the second author. The first author was a PhD student in Disability Studies, supervised by the second author, during data collection. The first author is a white bisexual person with chronic pain. The second author is a South Asian, cisgender, heterosexual woman without personal experience of chronic pain. Participants were aware that the interviewer was a fellow bisexual person with chronic pain, and some participants were aware of the interviewer’s membership in LGBTQ community organizations. Potential participants with whom the researchers had a personal relationship were not eligible. The first author used a reflexive journal, debriefing, and supervision to reflect on the possible influences of their positionality on the research process and ensure findings were grounded in participant experiences.

### 2.6 Ethics approval

This project was approved by the IRB at University of Illinois at Chicago, Protocol 2019-0740. All participants provided written consent.

### 2.7 Participants

Participants’ pseudonyms were Jamie, Roberta, Cindy, Angelina, KC, and Sabine. The sample included four cisgender women, one transgender woman, and one person who identified both as nonbinary and as a woman. One participant identified her race as Black/White/Islander, one as Asian, one as white/Hispanic, and three as white. Two participants were in their 20s, two in their 30s, and one each in their 40s and 50s. Three reported incomes below $25,000/yr, three above. Three reported their income was not enough to meet basic needs. Time with chronic pain ranged from three years to over 40 years, with four participants reporting pain lasting more than 10 years. All participants lived in the same large city in the United States.

## 3 Results

**Figure 2:**
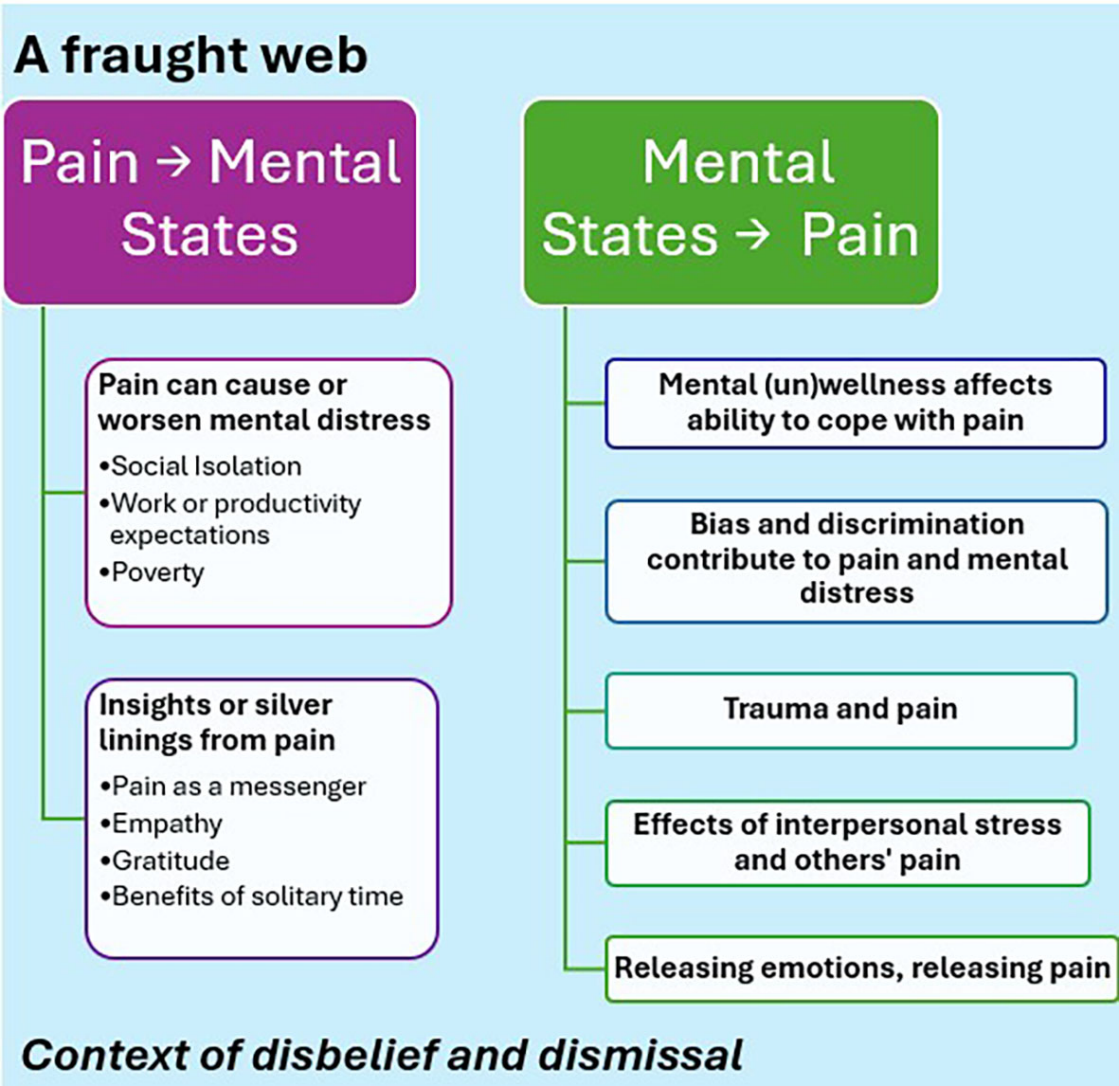
Illustration of relationships between themes and subthemes.

**Figure 2** illustrates the relationships between the three major group experiential themes and their subthemes. The first theme, *A fraught web*, describes the context in which all other themes and subthemes occur. The other two themes each describe a set of relationships, whereby Pain can affect mental states (group experiential theme two) and *Mental states can affect pain* (group experiential theme three) within this fraught context.

### 3.1 A fraught web

Participants described a complex “web” of relationships between chronic pain and the mind. This web was fraught due to the context of women frequently having their chronic pain dismissed as “all in [their] head[s]”(Jamie, Interview 1). Participants described multidirectional relationships whereby pain could affect their mental states, and mental states could also affect pain.

The metaphor of the web originated from KC (she/her or they/them). When asked about the relationship between pain and mental health, they explained,

“I don’t know if it is even just in one direction. I feel like it’s not even a direction. It’s probably like a web. That it’s all connected, especially related to trauma, anxiety, and depression…. They’re all related to pain and pain’s related to all of them and all of them can make each other worse,” (Interview 1).

Sabine (they/them) also experienced multidirectional relationships,

“A lot of my chronic pain journey has been trying to find the emotional roots of it. Because even though I know that, to an extent, a lot of it is physical stuff that’s wrong, it’s like—whether or not the pain came first or the emotion came first, they’re inextricably tied,” (Interview 1)

Later in the interview, Sabine elaborated,

“It’s like my perception of the pain is based on mental health, my mental health physically manifests as pain, [and] my ability to do the things that I like to do, or do the things that make me feel good, is impacted by physical pain,” (Interview 1)

This theme was complex and broad, encompassing differences between participants. Some participants, like KC and Sabine, openly described a variety of multidirectional links. Angelina (she/her) and Jamie (she/her) also described links in multiple directions between mental health and chronic pain, but they were very cautious to emphasize that any way in which mental health influences pain should not be used to dismiss the veracity of physical pain. For Roberta (she/her), pain simply made her mental health worse, but it could also contribute to a vicious cycle of low mood that made it harder to care for her pain. She also mentioned some links between trauma and pain in the abstract for other people. Cindy (she/her) mostly talked about pain making mental health worse, but she also saw relationships with trauma and discussed benefits of both emotional and physical release for pain. Cindy was very enthusiastic about the importance of this theme – in all its directions, including the opinions of other participants- at member checking. Although participants differed and sometimes disagreed, all their experiences taken together indeed illustrated a multidirectional web.

Many participants were cautious when discussing the topic of mental health and chronic pain because they had so frequently had their pain dismissed as “mental health only” (KC, Interview 2). Sanism is a form of structural oppression against people who are labeled as mentally ill (29). Sanism often intertwined with sexism contributed to dismissals of chronic pain. Angelina told me, “Now that’s a sore topic because (small pause), you know, doctors didn’t believe that I have fibromyalgia because I had to get a psych eval,” (Interview 1). Jamie said, “I feel like there’s this sort of bias towards, ‘This is emotional,’ which I think might be different if I were a man,” (Interview 1). Participants contended with the modern-day specter of the *hysterical woman*, a woman who is inventing symptoms to avoid work or gendered expectations (30, 31). Multiple participants evoked the word *hysteria* directly in discussing the dismissal of their pain (Jamie, Interview 1; KC, Interview 1). Once their pain was labeled as emotional or psychological, it could be swiftly disregarded as fake and thereby unworthy of care.

Despite her caution with the “sore topic”, Angelina did explain several relationships between pain and mental health,

“Well, mental health and physical health are connected, [ … ] they’re one in the same. And [ … ] if your body’s not right than your mind’s not right. Your mind’s not right, your body’s not right…. My fibromyalgia, it’s affecting my body … And chronic pain, it’s just, it saps the energy out of you for everything, you know? You just [ … ], you don’t want to move. [ … ] It’s just horrible. And [ … ] you don’t sleep. If you don’t sleep, you know, you’re never rested. And you never, your body’s not healing itself. And so, I think that’s the biggest thing for me as far as fibromyalgia … It stops me because … I’m never rested,” (Interview 2)

Jamie was extremely cautious in discussions about mental health and pain because of the context of disbelief and dismissal. She repeatedly emphasized that the relationships between mental health and pain are “inconsistent”, “not totally clear”, and that she wanted to “be really careful”. She made it clear to me that relationships between mental health and pain were not one-to-one or causal,

“I feel so mixed about it…. I do think that there is a relationship though. And I think it’s not always the thing [ … ] and I think that [ … ] the reason I’m so hesitant to talk about is because I think again, it gets over-applied. But I think especially if you’ve had most of your life without pain, and then all of a sudden you have pain. It’s devastating,” (Interview 1)

Jamie explained that the causes of pain are individual and multiple, and we should “never make an assumption about why someone is experiencing pain.” (Interview 2). In our member checking conversation, these contradictions arose again, when Jamie felt discomfort rereading her previous statement, “I don’t believe in purely psychological pain or purely physical. I just don’t think that exists.” (Interview 2). She was at a place with her pain where she felt she had pinpointed some clear physical causes and again was hesitant about the potential for any links between mental health and pain to be over-applied. She explained,

“I think that it’s so important to hold on to that [complexity] and to not try to oversimplify, because I think that’s where people end up getting hurt, is because it’s not quantifiable in the way that researchers want, it becomes then stripped down … so I think depicting the messiness is actually what research needs. [ … ] It’s not finding the answers- It’s naming the complexity and weaving that complexity [ … ] into patient care,” (Member Checking)

The preliminary review of first interviews highlighted a difference in participants’ comfort with interview questions about mental health, and their perceptions of the relationships between mental health and chronic pain. After KC and Sabine responded openly and comfortably to questions about mental health where others had been more cautious, the researcher asked them about their comfort with the topic. They readily identified the stigma that they believed would make other people less comfortable speaking about mental health, co-constructing the group experiential theme about the fraught context of dismissal, “If you’ve been fighting a long time to have your pain recognized, admitting to a mental health condition, I think, can be an extra struggle. (Sabine, Interview 1)”. KC interjected, “Oh, so it’s probably very triggering for them,” (Interview 1). KC and Sabine also theorized that perhaps they were more comfortable with the topic because they had worked in mental health settings, although both Angelina and Jamie were also mental health workers.

Participants were careful to say that their experiences of links between mental health and chronic pain were not universal – fearful that their words could fuel sanist or sexist ideas about psychological or hysterical pain and negatively impact credibility of other bi women with chronic pain, “I would not apply most of what I’m saying to a lot of the people that I’ve worked with or assisted on their pain journeys,” (Sabine, Interview 2). There are relationships between pain and the mind, but these connections do not mean that chronic pain is not real.

### 3.2 Pain can affect mental states

Pain could have numerous effects on participants’ mental states. Although the ways in which pain worsened mental distress were by far the most salient, participants also described some positive mental effects of pain. Pain could worsen mental distress via its associations with social isolation, work and productivity expectations, and poverty. On the other hand, sometimes silver linings of pain experiences included pain serving as a messenger, or encouraging empathy, gratitude and the appreciation of solitary time. These subthemes are illustrated in **Figure 2**.

#### 3.2.1 Pain can cause or worsen mental distress

All participants discussed ways that their chronic pain could cause or worsen mental distress. Sometimes the experience of pain itself contributed to mental distress. Jamie spoke about how pain could contribute to feelings of “despair.” She explained, “If I’m in a good headspace it’s probably because I’ve had some better days…. Versus if … I’ve had a lot of debilitating pain several days in a row, I’m starting from a less hopeful place,” (Interview 1).

Cindy spoke extensively about how, “in a lot of ways [her] emotional state [ … ] plays second fiddle to [her] bodily experience,” (Interview 2). She explained,

“If I’m in a bad mood, it’s not necessarily because [ … ] something in my life is making me upset necessarily. But [ … ] it usually stems from pain and any sort of mental stress I have usually has a physical symptom…. Or physical root. Moreso, to be honest. Like the pain is [ … ] the main issue and my mood is a side effect,” (Interview 1)

Cindy felt pain “must be dealt with first” before work could be done on mental health. This was illustrated by Cindy’s own conversation patterns in our interviews. In the first interview, the interviewer (first author) used nearly all question probes to encourage her to share in more depth, but Cindy spoke more easily and openly at the second interview. Cindy remarked on this change directly. She explained,

“I was in a lot more pain in October [at the first interview] than I am now. Or I guess maybe I’m in the same level of pain but I’m just better at dealing with it. And I’ve been able to be a lot more vulnerable and [ … ] doing a lot better work in therapy and feeling a lot happier in general since October just because my pain has been more manageable. So, I’m talking more personally as a result,” (Interview 2)

Her pain becoming more manageable – either through physical pain reduction or through better coping strategies – made Cindy more able to be emotionally open. Cindy’s spontaneous reflections on her response patterns contributed to the co-construction of this group experiential subtheme, which had also been an important personal experiential theme for her. She used her own behavior as a concrete example of how pain often changed her mental health and ability to function. In addition to how pain itself could cause mental distress, the effects of pain on other areas of participants’ lives could also contribute to mental distress. Chronic pain could worsen mental distress by contributing to social isolation or straining social relationships, by affecting participants’ ability to do valued activities or work, or through its cyclical relationship to poverty.

##### 3.2.1.1 Social isolation

Chronic pain often contributed to social isolation and this isolation could affect mental health, as was the case for Cindy:

“Chronic pain indirectly has caused a lot of isolation for me, and isolation affects my self-esteem, so indirectly that chronic pain definitely created self-esteem issues for me,” (Interview 1)

Social isolation was a major theme for most participants, although not all of them discussed it as directly linked to mental distress. For Jamie, the ways that her injury changed her ability to participate socially were emotionally painful to the point that Jamie felt a loss of identity, as if she was not herself at all, merely a “robot” or a “shell” of herself. This change was acutely emotionally painful and contributed to social anxiety that could worsen pain.

For Roberta, activity limitations were the central pathway through which pain worsened mental health. This affected her socially as well, because pain made it more difficult to do the activities that she would like to do with her friends. Roberta mentioned that her friends were very physically active (“marathon running friends”) and she hid her pain from them. She also believed that her friends might not even want to spend time with her due to her physical activity limitations. She explained, “It’s just really, really bringing me down, because my pain is not allowing me to be active. And not being active is allowing [ … ] my relationships to suffer. So, I mean, [ … ] it’s a vicious cycle,” (Interview 1).

##### 3.2.1.2 Work and productivity expectations

Chronic pain significantly affected participants’ ability to participate in valued activities and work. Jamie explained, “I mean, it’s heartbreaking … It was really sad for me to lose function and lose capacity and lose my identity because, again like I think I mentioned, so much of my identity was on production,” (Interview 1).

Sabine similarly discussed how development of chronic pain required one “to recalibrate all of your expectations for yourself, and that is a lot of work,” (Interview 1). Roberta experienced shame when she compared her current level of productivity to her former working life,

“As a teacher I had three jobs. …The fact that I went from that to, like, nothing (small laugh) really impacted me for sure, and it’s definitely a reason that’s driving me back to the classroom, I just do not want to be so lethargic, I feel lazy, I feel gross,” (Interview 2)

Sabine described having difficulty finding ways to accept their need to rest. They told me, “Rest is like the one thing that you’re not supposed to have in our society. It’s what makes you worthless” (Sabine, Interview 1).

##### 3.2.1.3 Poverty

Pain could contribute to financial precarity, and this precarity could engender severe mental distress. When I asked Sabine how the pain affects the way that they see themselves, they replied,

“I’ll preface this by saying, I’m okay now, like, I’m sturdy now. But [ … ] I’ve had multiple suicide attempts since the pain started. It makes it really difficult to believe that you’re gonna be able to make it work. The amount of times since the chronic pain started that I have been homeless or next to homeless is a lot….Yeah, it disrupts a lot of life and it makes it difficult to believe that it gets better or whatever,” (Interview 1)

Angelina, who had an income under $10,000 that was not enough to meet her basic needs, further explained the relationship between poverty and mental distress,

“Poverty and mental health go hand in hand because if you have poverty, you’re suffering from depression and anxiety because you don’t have food. You don’t have a way to pay for [ … ] your rent,” (Interview 2)

Cindy also reported an income under $10,000 per year and spoke of how uncertainty about meeting her basic needs affected her mental state, “I struggle a lot with feeling invisible for [ … ] the physical struggles that I’m facing and very frustrated and often scared because a lot of my bare minimum needs are not being met,” (Interview 2).

For Angelina, the cycle of poverty and mental distress could also feed back into pain. “We don’t really have any money. So that’s a big thing stress-related. And then [ … ] I have been having [ … ] neck pain [and] a lot of pressure in my head,” (Interview 2).

While the impact of chronic pain on mental health was mostly negative, participants did also describe some silver linings of their pain experiences.

#### 3.2.2 Insights or silver linings from pain

##### 3.2.2.1 Pain as a messenger

Several participants felt that their pain provided them with insights about themselves or about the world in general. Jamie sometimes viewed her pain as a messenger and explained that her pain “made [her] have to listen” where she “wasn’t listening before” because she was “so attuned to others,” (Interview 2). She further explained, “So I think of pain as actually like a protector, you know, and how we can really listen to it to [ … ] inform kind of the decisions that we make to take care of ourselves,” (Interview 2).

Sabine similarly sometimes viewed their pain as a messenger. They felt that “The body has a lot of wisdom and tells you when it’s had enough,” (Interview 1).

“This pain is there to remind me of [ … ] how to set boundaries for myself, and how to- I don’t know- it’s something that I struggle with is how to be grateful for it in some ways…. Because it calls to attention a lot of things that have been harmful or hurt that I tried to ignore….it’s like a reminder to take space for myself, not to stretch myself too thin,” (Sabine, Interview 1)

In our second interview, Sabine offered a caution about this perspective. They shared how sometimes people with pain or people who’ve experienced trauma can be blamed for their situation and asked to account for what they did to “deserve” the pain,

“I know that I talk about it a lot in terms of it being a message or a call, you know, there’s a narrative around pain that can be helpful that I use, but also at the same time, sometimes bad shit just happens. (laughs) You know, sometimes your body just breaks and there is no meaning behind it. And that’s really hard for people to deal with and accept,” (Interview 2)

Cindy found that her pain increased her awareness of her own body and explained, “I use pain as an opportunity to [ … ] become knowledgeable in regards to my own health mentally, physically, spiritually, whatever,” (Interview 1). Pain represented a sign to slow down, a teacher about the self, and even a lens through which to view the world.

KC drew direct parallels between the awareness pain conferred about the body and the awareness marginalized ideas, including disability, confer about the world,

“When I experience pain, it’s [….] always reminding me that my body is there, I’m always experiencing my body … It’s not a perfect analogy, but in the same way that people who have a dominant identity [ … ] may not fully experience their identity because it is the default or because it’s just the accepted norm. They’re just like, “Oh, [ … ] I’m regular” Or, “I’m normal.” Whereas for myself, with my marginalized identities, [ … ] I’m almost always experiencing those … the same way that when I’m in pain, I’m aware of the fact that my body is there and that I’m in pain,”

The pain of the body was described in parallel to the pains of structural oppression. People with marginalized identities have experiences that make them acutely aware of the workings of systems of power. Participants’ pain offered insights on pain both within and beyond the body.

##### 3.2.2.2 Empathy

All participants mentioned that their pain increased their empathy for other people in difficult situations. Cindy explained,

“When my chronic pain is most severe, I felt [ … ] very emotionally desperate or [ … ] financially or just [ … ] generally unstable. [ … ] So, I sort of take that knowledge with me when I interact with other people and knowing that everyone has their own unique situation that I cannot fully experience or comprehend. And that [ … ] despite of any advantages they may have or disadvantages they may have [ … ] that at their core that [ … ] I can see myself in them, and I can see my own struggles within them and there may be something I can provide, “(Interview 2)

Jamie spoke about how her experiences with chronic pain and chronic illness helped her question snap judgments about other people. KC explained that their pain and chronic illness disrupted ableist thinking and patterns and made them into a less ableist, more compassionate person.

Roberta agreed about pain fostering empathy, and Angelina shared how her life experiences including trauma, poverty, and chronic pain inspired her to work in social justice and social services. Angelina was very involved in a nonprofit organization supporting bisexual people in person and online.

##### 3.2.2.3 Gratitude

Some participants explained that experiencing pain made them more attuned to, and grateful for, small moments of joy in life. Jamie explained,

I think being a person with chronic pain. I have learned to really appreciate the little things, like walking down the street and not losing my balance or just seeing the beauty in everyday things. … if I do recover [ … ]-- or as I recover, I don’t want to lose that part of me that just feels so lucky to be alive. (Interview 2)

Cindy felt a greater appreciation for positive moments in her life, “maybe I cherish the moments that are more that are [ … ] more stable more as a result of pain. I would say that I work very hard, much harder to gain those moments as a result of pain,” (Interview 1).

##### 3.2.2.4 Benefits of solitary time

Although changes in their ability to leave the house and socialize could engender role changes and mental distress, some participants also reported a great deal of enjoyment in their solitary, at-home activities. Angelina and Jamie talked about using their time at home to connect with others by talking about favorite TV shows. Cindy talked about how “being isolated gives you a lot of time for introspection. Or being sedentary gives you a lot of time to use your imagination,” (Interview 1). A musician herself, she personally knew a lot of other queer and trans women musicians with pain and suggested that having so much alone time due to pain gave them time to perfect their crafts.

“If you have chronic pain, you have a lot of free time because you’re stuck alone or whatever…. When [ … ] I was much younger I would do one hour to eight hours of practice a day on [instrument] because that was just how I got through the day … And that’s how I got really good,” (Interview 2)

KC reported that since childhood, she had a lot of solitary hobbies and could be alone without feeling lonely up to a point where “[if] I’m alone too long then I do feel lonely,” (Interview 1)

### 3.3 Mental states can affect pain

Just as pain affected participants’ mental states (mostly by engendering mental distress), the converse was also true where mental states could affect bodily pain both directly and indirectly. These relationships included the ways in which mental (un)wellness affects one’s ability to cope with pain, bias and discrimination contribute to pain and mental distress, trauma and pain are intertwined, interpersonal relationships can affect one’s pain, and releasing emotions can be related to releasing pain. These subthemes are illustrated in **Figure 2**.

#### 3.3.1 Mental (un)wellness affects ability to cope with or care for pain

Although participants’ pain did not go away when they were feeling mentally well, being in a good mental state could sometimes make it easier to cope with the pain. Jamie explained,

“It’s not always totally clear for me because there might be days where I’m [ … ] feeling so hopeful and excited about life and I’m experiencing a lot of pain. So it doesn’t always seem to be [ … ] causal but I do think that it makes the pain easier to manage when I’m in a good headspace … I feel like I’m often walking this line of despair and hope and it’s kind of like it’s this tightrope, and … I think pain [ … ] can kind of bring me into this place of despair. But I think if I’m starting at a place of higher hope that day, despair is a little bit further away,” (Interview 1)

For Angelina, doing enjoyable activities could help take her mind off the pain,

“I think when I’m happy, or I’m gonna get out and do something extra … you know I’m going to a movie, we going to dinner … I’m still in pain. I just kind of don’t notice it as much. But [ … ] it’s definitely there because when I get up and go to the bathroom, I can barely walk,” (Interview 1)

Sabine also stated that mental wellness could help them better manage their pain, and that they needed to plan around this, when possible, for example by avoiding workplaces where they were mistreated. KC explained that mental wellness and social supports could help buffer the possible painful effects of sexist experiences like street harassment.

“When I have had more [ … ] social supports and more quote unquote “protective factors” in my life, the things that go wrong, I can kind of brush [sexist experiences] off, and they don’t bother me as much as when I am already in a really traumatized space,” (Interview 1)

Although mental wellness didn’t erase pain, it could decrease its impact or make it easier to manage. Roberta and Cindy spoke less directly about how mental wellness might make it easier to cope with pain. Both women mostly emphasized the negative effects of pain on mental health. However, pain could also contribute to a “catch-22” for Roberta, whereby pain made it difficult for her to be physically active, which contributed to her feeling poorly about herself and not engaging in self-care. This fed into a cycle of lower activity and lower mood that could also worsen pain.

Cindy explained that when she is feeling mentally distressed– at least in part because of her chronic pain which is at the “root” of her mental distress – she can feel nihilistic and overdo her daily activities in such a way as to worsen her pain.

“If I’m feeling nihilistic and just like, “Well, doesn’t matter, I’m just gonna push myself to my limit regardless because it doesn’t matter if I don’t or not.” And [ … ] (quick sigh) I would see that being a mental health issue as a side effect of chronic pain. (Interview 1)

So, she wound up in a cycle, like Roberta, whereby pain worsened her mental distress, and when she was mentally distressed, she was sometimes less likely to care for her pain.

#### 3.3.2 Pain and mental distress as reactions to bias and discrimination

Political factors and experiences of discrimination or bias could influence both mental health and chronic pain. Several participants described how the political environment under the first Trump administration could increase their mental distress. This mental distress in turn sometimes contributed to increased pain. KC explained how the intensified persecution of minorities affected her physically and mentally,

“I know that my pain, I know that my stress has really, really rapidly increased, like exponentially increased over the past [ … ] three or so years, just because of the social and political moment that we’re in. You know, things were not excellent before for a lot of the communities that I’m part of … but [ … ] there’s just something about [ … ] the past three years since Trump got elected that have been especially awful and terrible. And I think some of it is like the stress and the fear [ … ] of what’s happening right now. And also, just the feelings of despair and hopelessness, that it can engender as well,” (Interview 1)

Jamie described this political hopelessness as painful,

“In my own body [ … ] every day I feel helpless to some extent. Which I think mirrors the current political landscape, you know, it’s like, I feel so helpless. And I think I feel especially helpless as a person who has physical limitations who can’t, literally go to the border and offer my skills … I think that it’s painful,” (Interview 1)

In our member checking session, Jamie noted that it was interesting how much she used the word painful or pain to describe other areas of her life influenced by pain versus strictly the physical pain itself. Pain was not only a bodily phenomenon caused by the world, but also a lens through which to see the world. Beyond being a metaphor for social phenomena, pain was social and political itself, an experience that could not be confined to the physical body. KC also drew a parallel between the stressful political situation in her country of birth and her pain,

“I can’t really explain why but I know that when there [are] a lot of stressful things happening related to [the country where I was born], I feel that deeply in my body … just the amount that it’s been in the news and fear of [ … ] what is this current administration going to do? Am I going to wake up tomorrow and the country that I was born in will have been bombed off of the land that it’s attached to? Yeah, I feel that deeply and that’s a really specific kind of stress and pain but feels very similar to my fibromyalgia pain,” (Interview 1)

Political stress and fear were embodied and could create both mental distress and pain. Angelina and Sabine discussed how the news affected their bodies. When asked about, “things in the environment that affect your pain” Angelina’s immediate response was about politics.

“(Immediately). Trump. [ … ] I’ve been starting to get really a lot of headaches. And I’ve been really stressed about him that [ … ] we cut cable … And it’s weird, I can’t see my news. I was like news news news … I was watching too much of him,” (Interview 1)

Some participants also described ways that identity bias, microaggressions, or discrimination could affect their pain. As KC discussed above, street harassment could be mentally and physically painful. When we were discussing things that helped with their pain, Sabine excitedly told me about an experience they had at a Burning Man (arts and music festival) event,

“I was there with two other people with chronic pain. And all of us had the best chronic pain week that we had had. …. I just felt good. And I felt safe … One of the people I was with too … walking is difficult for them. And one of the reasons is because of where they live, they get catcalled all the time. And I remember them walking, all the way around the lake, and then being like, “This is strange. This is the first time I’ve ever been able to just walk outside as myself. And nobody said anything to me.” And having that freedom and feeling that safety of just being able to show up authentically and exist did a lot for all of our pain,” (Interview 1)

Roberta recounted similar experience where microaggressions and bias stemming from fatphobia affected her ability to perform physical activities,

“On my worst [ … ] emotional mental health day, I feel like people look at me and they’re like, “Ew. Gross” (laughs). And they just don’t know what I’m going through. They’re like, “Well, this person should just get off the couch and move,” And, going up stairs, going downstairs…. Sometimes when there are people around, I can’t maneuver those things as well.” (Interview 1).

Fear of fatphobic judgments could make public physical movement more difficult for Roberta, which could feed into the cycle of inactivity and pain that so distressed her. Angelina described instances in which witnessing racism exacerbated her symptoms. She described her response to a video that was shared on social media of someone going on a racist tirade,

“Just screaming, calling them the N-word. I mean, like 20 times, “I hate you. [ … ] If I could do it, I would kill you.” That’s what she said … That irritated me, [ … ] my headache grew bigger and I was having panic- like I’m talking about it right now. And I just felt like my back, it’s bothering me and I’m having pressure right here. And I don’t even have a cold or anything. (Laughs) So those kind of things bother me. And a lot of racism right now. It’s really bothering me,” (Interview 1)

Anti-Blackness was an embodied experience that affected both body and mind. For Cindy, discrimination influenced the creation of pain through trauma that was disproportionately experienced by marginalized people. She believed that “childhood trauma really influences the creation of chronic pain”. She elaborated,

“So, when there’s emotional stress, physical stress is created and that physical stress creates maladaptive growth, which creates long term problems. And the way in which women and [ … ] queer people are treated in society is negative. So as a result, there’s a lot of negative emotions that are confronted when you’re young. And that would be my general perception of the queer women who I know who have chronic pain. Not all of them but quite a lot of them. Feeling marginalized from a young age,” (Interview 2).

Bias and discrimination also had social effects. Angelina experienced exclusion from the bi community because of ageism, ableism, and racism. Cindy discussed how sports – which she felt could be very helpful for chronic pain – were sometimes not accessible due to cissexism or heterosexism. KC and Sabine described exclusion from the queer community because of binegativity, which contributed to social isolation. These social effects could sometimes be distressing. Sabine explained how binegativity in social settings could worsen their physical symptoms,

“It depends on which part of the queer community you’re in, but biphobia is real. And [ … ] trying to date people gets weird. I mean, which also intersects then with chronic pain and chronic illness, where it’s like, what do you have the energy for? Because you spend most of your day out in normal society, when you go into [ … ] a space that’s supposed to feel good, but instead is a space where you’re [ … ] fighting for who you are, it just is very tiring … Get a tummy ache, leave the party,” (Interview 1)

Overall, politics and identity bias affected participants’ mental states and their pain.

#### 3.3.3 Trauma and pain

Relatedly, traumatic experiences also influenced participants’ mental health and their pain. KC experienced multiple kinds of pain, in addition to other chronic conditions. Speaking about different pain experiences across her lifetime, she described that the pain she experienced after an accident as a teenager “felt very physical” and did not seem to have mental trauma attached to it. In contrast, her adult pain was “more related to how [her] body has been able to process or not process trauma” (KC, Interview 2), and therefore coping with her adult pain had required coping with trauma. KC was diagnosed with fibromyalgia soon after experiencing sexual violence. Although other factors also contributed to her pain around this time (the weather, other symptoms like fatigue and brain fog, getting back into sports) – she highlighted the trauma as very tightly associated. KC believed that if she had not experienced that trauma, she likely would not have chronic pain. In addition to experiences of sexual violence, KC also identified generational trauma as an important factor in their eventual development of chronic pain. They explained the trauma that they believed their biological parent must have gone through in deciding to give a child up for adoption, then their early experiences of foster parentage, and transnational adoption.

“So, probably those two pieces, you know, the generational trauma and not being able to unravel that, and then the sexual violence I think are probably- if I had to diagnose myself, which my doctor hates when I do- I would say that those are probably the two [ … ] biggest indicators of me eventually ending up with multiple chronic conditions, one of which includes chronic pain as a symptom,” (Interview 1)

Cindy similarly felt that childhood trauma could contribute to chronic pain for queer women. Experiences of sexism and heterosexism could cause mental distress. Speaking about developing pain in childhood, she explained,

“When the earlier that pain develops, the harder it is to sort of process and the less support there is, the harder it is to advocate for. So, it creates a system in which people are left to fend on their own and create their own narrative. And that can often create a lot of bad, bad science or bad, bad malformed coping mechanisms to make up for the lack of support,” (Interview 2)

She told me that therapy in adulthood was helpful in working through this, but that addressing the physical root of her pain was also essential.

Sabine also experienced their pain as linked to trauma in the form of interpersonal violence. When I asked them how relationships affected their pain, they explained that traumatic interpersonal events could affect both their mental distress and their pain. Speaking about traumatic events that happened a few years ago, in the same year that they developed one of their chronic pain conditions, they described several instances of intimate partner violence. Details about the violence and Sabine’s bodily symptoms have been anonymized.

“[Describes relationship conflict involving their queer and polyamorous identities] … that was like some deeply ingrained trauma that then got—the reason that I ended up being able to cut off that person was that-- and I mean, you can even see while I’m talking about this, my [physical symptoms are becoming visible]-

Interviewer: Yeah, and we can stop talking about it anytime that you want to.

Sabine: Yeah, but it’s like, so these things are all related. [Describes another instance of dating trauma with a different partner] … So all of that happened, I cut off a bunch of people who I had perceived as being part of my core community at around the same time [and] that was like within a few weeks of the [chronic pain condition] getting very, very bad….I mean, that ties into being a woman, that ties into being a bi woman, because sexual assault, sexual violence, gendered violence are all more likely if you’re bisexual because you’re perceived as deviant and less human,” (Interview 1)

Trauma seemed to be linked to pain both as a physical manifestation of the emotional trauma, and because fleeing violent situations led them to make impulsive decisions (like moving, undergoing elective surgery, cutting off community) that contributed to acquiring new impairments or losing social support. Thinking about the trauma again could make their symptoms flare. Later in the interview when I asked them what questions they would like researchers to ask about bi+ women with chronic pain, their personal experiences seemed to inform a research interest and hypothesis relating trauma and pain, “I’m interested in how sexual assault and sexual violence, specifically in the bi community, if that connects at all to chronic pain in the bi community. Because I’m pretty sure there’s a connection in there somewhere,” (Interview 1).

Like Sabine, who described a year wherein several traumatic events occurred and their pain began or intensified, Angelina described several discrete years in her life wherein multiple traumatic events clustered together. She returned to three years in her life multiple times in the interview- a year in pre-adolescence, one in early adulthood, and the year before our interview. One of her pain conditions was described as beginning in the pre-adolescent year wherein she experienced multiple instances of childhood trauma. Another pain condition began around the year in adulthood wherein she had a serious physical injury and a separate near-death medical experience.

In one interview, which was conducted via videoconference, one participant described memories of specific traumatic experience she survived as an adolescent that had been haunting her in past days due to a recent experience that reminded her of that trauma. She requested that her pseudonym be withheld when discussing this topic because of current intimate partner violence. The participant was in a private room with the door closed during the interview, but we used the chat (rather than speaking aloud) as an extra confidentiality measure when she discussed her partner directly. The interviewer checked in with the participant after interviews about her safety and offered resources. At member checking, we discussed how best to anonymize her story to safely share her experiences. She explained how traumatic experiences could trigger pain,

“It’s a very traumatic thing that [ … ] keeps on resurfacing, especially when I’m going through this kind of stuff. Like when I’m having pain or when I’m like [ … ] trapped, or I can’t move or, you know, it’s kind of kicks in then. But so it’s been kind of strong right now lately cuz this weird stuff happening,” (Interview 2)

Pain could surround the original traumatic events but could also trigger memories of past traumatic events.

Although Roberta did not talk about a relationship between trauma and pain for her personally, she did think there was a connection between trauma and pain on a community level. Echoing KC, Sabine, and Cindy, Roberta acknowledged that queer people may disproportionately experience trauma, and perhaps this could be associated with chronic pain.

Jamie had extensive professional training about pain and trauma from her work in the mental healthcare field. As she did for many issues of mental health and pain, she offered a caution that the relationships between trauma and pain are complicated,

“In [my specific mental healthcare training], it’s like, “all pain is trauma.” [ … ] And I’m like, “Well, no.” [ … ] I think pain is more complicated than that. [ … ] And on one hand, I could think, “Okay, well, I [experienced a physical injury], and I have an incomplete self-protective response. So that’s why [ … ] when I am out in the world, my pain pattern is literally where I [experienced my physical injury] because something is trying to work itself out. And if I were able to create space to just work that out and just process the trauma, then I would be pain free.” Which is literally a lot of my learning. You know? Which I think could be amazing if that were the case. And it hasn’t been the case for me. That hasn’t been the magic bullet for me,” (Interview 2)

Participants described a variety of ways that trauma could be linked to chronic pain on the personal and community levels for bi+ women. These relationships between chronic pain and trauma had commonalities across participants, but they were not identical.

#### 3.3.4 Effects of interpersonal stress and others’ pain

Several participants discussed how stress or conflict in interpersonal relationships could contribute to mental distress and pain.

One participant discussed how her and her partner “argue[d] almost every day” (Anonymized Participant, Interview 1). Pain, illness, and disability were the most common topics of arguments in her relationship. Her partner also had chronic conditions and they “always argue about whose pain is worse,” (Interview 1). The arguments and intimate partner violence could also worsen pain.

“When I have arguments with my [partner], my pain aggravates and that’s really bad. We argue almost every day sometimes. (laughing) … when we’re not talking then that aggravates it a lot,” (Interview 1)

KC similarly explained, “I’ve had relationships where the stress of those relationships has exacerbated my pain,” (Interview 1).

Sometimes relationships affected participants’ pain because of the ways that participants could pick up on others’ intense emotions. Jamie said,

“When there’s stress in my relationship, you know, [ … ] I think that stress can impact my pain and I’m very, very empathic, you know, so while I try to work on, my physical energetic boundaries, sometimes it’s just like I take a lot in. So I think that’s a biggie,” (Interview 1)

Sabine spoke about this attunement to others’ trauma and emotions as particularly relevant for people in helping professions. This was a pattern Sabine had experienced in their past work and witnessed through being close with people working in social service professions. Sabine explained that almost all their friends had chronic illness of some kind and felt this was perhaps because they gravitated toward compassionate people. In our second interview, I asked whether pain makes people compassionate, or compassion creates pain. Sabine explained that they thought it could happen both ways and gave examples of both directionalities from their friends.

While analyzing the first interviews, the first author realized that most participants worked in social service or caring professions and/or were involved in social justice work. They asked a follow-up question in the second interviews about how pain might affect participants’ social service or social justice work, and/or how this work affects their pain. Cindy analyzed the complexity of the possible relationships,

“I really honestly hate but also love the fact that I have been predisposed to do care work. I really hate that having chronic pain makes care work long-term impossible. But [ … ] I also hate that having chronic pain makes me predisposed to being empathetic to people in need. And [ … ] makes me more emotionally mindful. And [ … ] more suited to meeting someone who’s in need. But on the flipside, I do like caring for people. It’s very rewarding. When it becomes an obligation, [ … ] it becomes sour for me and it is something I do not want to do for work ever again. My body is not able to sustain it. My mind, my mental health [ … ] will become unstable because I’m putting someone else’s needs above my own, ultimately. Because that’s kind of what work is. To me at least, it throws my self-care regimen out of whack. But, I mean, helping people is one of the most fulfilling things I can do in moderation. It’s really a healing experience,” (Interview 2)

When the interviewer asked Jamie this question in the second interview, they prefaced it with the finding that many of the participants worked in social service or caring professions. Before they finished asking the question, Jamie interjected, “I don’t think that’s a coincidence,”. She explained some possible connections between care work and pain,

“So, I want to be really careful about this because I do not want to blame myself or anybody else, but I think some of what I was talking about in terms of pain being something that’s actually made me have to listen is that I wasn’t listening before. You know? Because I was so attuned to other people at the expense of myself … Maybe that’s not fair. But [ … ] I feel like-- at least for me and my situation--there’s this way in which I was so focused on others, both in my personal relationships and in work, in a way that I was neglecting myself. And I don’t know how that has impacted [ … ] how I’m doing now. Or how my pain is. But I do now view my pain as a signal. A way to know how and when to take care of myself,” (Interview 2)

Care workers, like most of the participants, could prioritize others to their own detriment, and pain could serve as an alarm about this imbalance. This attunement to others could also promote the absorption of others’ pain into participants bodies. The body of the woman in pain is porous^4^, both influenced by and influencing the people and environments around them. For Sabine, the ways that others’ emotions could affect pain were tied to gender. They explained their understanding of the “cultural archetype” of womanhood,.

“Almost in a historical sense … When people talk about womanhood, they often talk about pain, and in reference they’re sometimes talking about the pain of childbirth, but I think a lot of what they’re talking about is the way in which, that societal role has put us into this receiving position. So, we’re constantly receiving all these emotions and feelings, and we’re taking all these things in, and those things become illness and pain. And … I think that being a woman in this society means that you take everything in and you let it hurt you. And that that can manifest itself in an illness, or in a physical pain, or you know, a variety of inappropriate ways to dispense rage,” (Interview 1)

They also continued:

“The feminine ego [ … ] receives and takes and embraces that harm when there also needs to be a part of the self that lets it go. But we don’t have like cultural practices that do that anymore. Not really. There’s no rituals to let go of that pain, there’s no community with which to disperse it between us [ … ]—we all just kind of take it, and then –with nowhere to go, it figures out how to be expressed,” (Interview 1)

Sabine, Jamie, and Cindy explicitly talked about the negative effects of holding painful emotions in. As Jamie explained, “I think often if we don’t have an outlet for what’s happening, again, we can hold certain things,” (Interview 1). Because holding painful emotions in could be harmful, these participants also discussed the importance of finding outlets for such emotional pain.

#### 3.3.5 Releasing emotions, releasing pain

Cindy recommended sports – particularly aggressive sports such as wrestling, martial arts, and roller derby – as helpful releases for women with chronic pain. She felt that aggressive sports could allow “negative emotions to be used in a constructive way” (Interview 2), which could be helpful to people with chronic pain. She advised that a “less extreme” way of getting this “relief” could also be yoga or massage for some people. Cindy also suggested that artistic activities could provide this helpful “emotional release” (Interview 2) for people with pain. Despite the helpfulness of sports and arts, Cindy explained that these activities were not always accessible to queer and trans people. She explained that sports especially could be inaccessible due to potential discrimination and violence.” (Interview 2).

Sabine discussed the importance of releasing pain in community. When I asked in our second interview for more information about their idea of dispersing hurt via social rituals, they offered the example of keening. Keening is a historical Irish mourning practice wherein a group of women would scream or wail together over the death of a loved one. Sabine described this as an example of a cultural ritual that could be very helpful for releasing hurt, but which is no longer widely practiced. Keening could be powerful because of both the release itself and the community support. Instead of “standing away” from someone in pain, loved ones “get in there and experience it with them to have a communal experience of like pain and rage,” (Interview 2). Thinking about other examples of release practices, Sabine thought of 90s club and rave culture wherein people would release emotions in community through dancing, music, alcohol, and drugs. They thought that gay clubs could theoretically offer this release but mentioned that lesbian bars had disappeared all over the country^5^, and gay bars oriented toward men did not feel accessible or safe to them. Burning Man events allowed them some of this release by creating a free space to be “surrounded by people” and “do what you need to do or feel what you need to feel,” (Interview 2).

Jamie spoke personally about the benefits of externalizing emotions and pain when it came to sharing about mental distress with loved ones. In the abstract, she also thought about the possible effects of being unable to be open about one’s identities.

“I do think that [ … ] usually when there are ways in which we feel like we can’t express ourself, [ … ] there is a physiological constriction that happens, whether it’s our gut gets crunched, or [ … ] our shoulders get tight, or whatever it is, I think there’s often, this way in which we’re holding on so strongly, that happens both psychologically and physically. And so, I think that there can be relief and release when you start to externalize some of those pieces, or [ … ] like, what’s coming to me is, find a home for them?” (Interview 2)

For Cindy, Sabine, and Jamie, pain could sometimes be related to personal truths or difficult emotions that one held in and felt unable to release. Roberta did not speak about emotional release as related to pain. Although KC and Angelina did not discuss emotional release as personally or in-depth as Jamie, Cindy, and Sabine did, they alluded to this approach. Angelina explained that when she was down, she liked to watch a sad movie and really cry it out. Like Jamie, KC explained that emotional release – like all other aspects of the fraught web relating chronic pain and mental health- could be one part of the puzzle but could not tell the full story.

## 4 Discussion

Participants described their bodily pain as both affecting and affected by their mental states. These relationships were too complex to summarize in linear terms. They were often multidirectional, partial, messy, and contested. This web of complex relationships existed in a context that was highly fraught.

Sanist epistemic violence was the primary reason that participants viewed the web as fraught. People with chronic pain experience pervasive disbelief and dismissal of their knowledge claims. Such disbelief and dismissal can be considered “epistemic violence”, which is defined as an injustice by which, “certain persons or groups within society are disqualified as legitimate knowers at a structural level” (37, p123). In health encounters, people in chronic pain are often striving to have their stories and their pain believed to obtain needed medical care (38, 39). When their pain is not found to have a biological cause – or even if it has a biological cause but the patient is deemed to be overly distressed – patients in pain are often sent to a psychiatrist instead of a pain specialist (40, 41). Receiving a psychiatric diagnosis may lead to further epistemic violence that makes it harder for the patient to have their pain believed in the future (37, 40).

Participants already faced epistemic violence based on their sexuality, gender, and other marginalized identities – so the potential for dismissal engendered by being labeled or perceived as “mentally ill” could be devastating. The existence of links between mental health and chronic pain does not invalidate the veracity of women’s claims of chronic pain or their requests for social, medical, and structural care and accommodations. Modern biomedicine’s division of specialties based on body systems, and a strict separation between the ‘physical body’ and the ‘mind’ (42) has sometimes led to disregard of mental distress that co-occurs with chronic pain (or vice versa). This web of relationships between mental health and chronic pain is deeply fraught and must be approached with a great deal of nuance.

### 4.1 Pain can contribute to mental distress

Feminist disability studies scholars bring attention to the importance of acknowledging the unpleasantness of some bodily impairments (43, 44). Whereas many early disability studies scholars emphasized the social aspects of disability to the point of neglecting bodily impairment, feminist disability studies scholars pointed out that we must also acknowledge the body (43, 44). Applying Crow’s work, Price writes that “when people experience pain, their experiences should be treated as real. And in some cases, as really bad,” (45, p276). This honest acknowledgement of impairment is especially vital because marginalized populations experience painful impairments at disproportionate rates (43). Indeed, participants often experienced their pain as deeply distressing and spoke of it as a bodily, rather than social, phenomenon.

In other cases, pain contributed to mental distress more indirectly, by affecting participants’ ability to engage in productive work, social interactions or desired activities. On one level, the physical experience of pain itself prevented participants from doing activities they valued in the ways they preferred. However, the activity limitations were often not simply caused by the pain itself, but also by ableism and inaccessibility. Feminist disability studies scholars also underscore the importance of considering the structural factors that often cause painful impairments for marginalized people (32), and the cultural discourses surrounding pain that make it more distressing (33, 46). Inability to work, for example, was often not distressing because participants missed the work itself, but rather because they felt their inability to work made them “worthless” by society’s standards, or because their inability to work caused them to live in poverty. TL Lewis defines ableism as a form of systemic oppression that is closely tied with other forms of oppression such as racism, sexism, and colonialism, which estimate a person’s worth based on socially constructed ideas of achievement and productivity (47).

Capitalist ideals that a worthy person is a productive person are foundational in Lewis’ definition of ableism. Ableism was felt deeply by participants. Roberta judged herself harshly for not being as productive and active as she had been in the past. Jamie and Sabine had to “recalibrate” their identities and expectations of themselves. In addition to the psychological effects of capitalist ideals of productivity and worth, participants also felt the material effects. For many, pain was a major contributor to living in poverty. The daily conditions of poverty were mentally distressing and further contributed to their pain or impeded their ability to manage it. A large mixed methods study in Canada identified four primary pathways through which poverty influences mental health for bisexual women (48). Experiences of childhood trauma, binegativity, and poverty contribute to adult poverty and mental distress. Second, experiences of workplace binegativity serve as a barrier to employment and financial stability. Third, discrimination based on both bisexuality and socioeconomic status affects access to community and social support. This lack of social support in turn contributes to mental distress. Finally, cost concerns and perceived bias impede access to mental health care (48). Each of the four pathways described by Ross and colleagues intertwined with pain experiences for the women in this study. Chronic pain intersected with other experiences of marginalization to contribute to poverty, mental distress, and continued pain for participants.

### 4.2 Insights from chronic pain

Although pain often engenders mental distress, it could also provide unique insights about the world. In addition to bringing awareness to the body, pain confers situated knowledge of norms and expectations. Feminist standpoint (49) and sitpoint (50) theories point out that marginalized people have unique insight into the workings of systems of power. Situated knowledge from chronic pain can help illustrate the contours of ableism, sanism, and other oppressions more fully. The boundaries between the individual body in pain and the broader world are permeable – bodies are both affected by and affect the people and environments around them (32–34).

### 4.3 Mental distress affects pain

Participants found that politics, discrimination, and trauma contributed to both mental distress and chronic pain. In some cases, a negative experience triggered both mental distress and chronic pain simultaneously. In other cases, the chronic pain seemed to appear after the initial mental distress.

Feminist Mad Studies scholars have argued that much mental distress and even behavior labeled as “mental illness” stems from the trauma of sexist violence (51–53). In such cases, they argue, distress is an expected and wholly reasonable response to the violence suffered, and psychiatric “medicalization of distress,” (53, p21) creates further stigma and trauma (51, 53). Similarly, Reeve theorizes that sanism and ableism can contribute to mental distress (54). Building on Anne Plumb’s work, Reeve explains the substantial effect of sanism/ableism on mental distress and argues that “society distresses us” (54, p110). The experiences of women in this study further illustrate how *society pains us*.

Within health equity research, the minority stress framework explains that experiences of stressors related to a marginalized identity contribute to mental distress and diagnosable mental illness (19). Bisexual+ women consistently demonstrate high levels of mental distress, even when compared to other sexual minorities, likely due to the intersecting effects of sexism, heterosexism, and monosexism (1). Research indicates that multiple types of discrimination (including ableist, sexist, heterosexist, racist, and binegative) are also associated with higher reported rates of pain (17, 55). A minority stress framework proposed by Lick and colleagues (20) could explain how minority stressors contribute to bodily pain. Lick and colleagues theorize that “sociocultural stressors” (including discriminatory acts, social policies, and healthcare barriers) contribute to both psychological and physiological stress responses. Once stress responses are created, they affect both health behaviors and physical and mental health status (20). The bisexual+ women in this study saw sociocultural stressors as related to both their mental and physical health and their ability to engage in necessary health behaviors. Minority stress is part of how these bisexual+ women made sense of their pain.

Within disability studies, Siebers’ theory of complex embodiment offers another framework for understanding how social stressors may affect bodies. Siebers suggests that we consider the social construction of identities as we would consider literal construction of a building (34). Consider, for example, the common stereotype of bisexual women as promiscuous and unfaithful. This stereotype is constructed in media and personal conversations and is a commonly held belief as documented by survey research (56). This stereotype can influence how bisexual women are treated. Recent research examined the link between partner jealousy and intimate partner violence among behaviorally bisexual people assigned female at birth (57). Researchers found that partner jealousy – which they theorize to be informed by the stereotypes of bisexuals as promiscuous and unfaithful – was correlated to intimate partner violence. An additional study found that people reading about sexual assaults were more likely to label bisexual victims as more promiscuous and more responsible for the assault (58). As participants in the present study described and past research indicates, intimate partner violence and sexual violence can contribute to pain (16, 59). In this way, representations of bisexuality in media and culture can predispose bisexual women to sexual and intimate partner violence, the trauma from which comes to be built into their bodies and minds as chronic pain and distress. In the present study, participants experienced sexual violence and intimate partner violence that was related to their sexuality, gender, and other marginalized identities. These experiences of violence were in turn associated with pain and interlinked mental distress for some of the participants.

Research suggests that people in chronic pain are frequently survivors of sexual violence (11), people with histories of intimate partner violence are more likely to have headaches or chronic pain (16), childhood abuse is linked to development of chronic pain (60), fibromyalgia diagnoses are associated with sexual and physical assault/abuse histories (59), and pain and post-traumatic stress disorder (PTSD) are tightly linked (61). A recent survey of LGBTQ people with chronic pain showed that people with a higher number of adverse childhood experiences reported higher average recent pain scores in adulthood (62). In the survey, history of childhood sexual trauma was found to be associated with higher risks of pelvic pain and fibromyalgia. In addition, transgender and gender diverse people reported higher rates of both adverse childhood experiences and pain than their cisgender peers (62). An analysis of common comorbidity of chronic pain and PTSD concluded that it is difficult to ascertain directional causal pathways between the two and that each can trigger the onset of the other as well as play a role in its maintenance (61).

Like the other mind body relationships described by participants, the relationships between trauma and pain form a complex web that resists linear explanation. Bisexual women experience high rates of sociocultural stressors, which can contribute to both mental distress and chronic pain. Such pain can be part of the embodiment of certain social identities; part of the way that social stress is written onto bodies (34). Thus, historical and political factors that create and sustain social stressors cannot be divorced from understandings of pain or suffering of marginalized individuals (32, 38).

## 5 Conclusion: toward a holistic approach grounded in belief

Participants’ experiences illustrate the complex ways in which systems of oppression intersect to engender a complex web of pain and mental distress of marginalized people. Their experiences also suggest that pain could be a messenger, signaling the need for change at individual and structural levels. The suffering of bisexual+ women should inform both collective healing from trauma that has already been created (e.g. through social support and access to healthcare and resources) as well as collective action to prevent future harm (e.g. by transforming unjust policies and systems). Collective healing requires identifying oppression, taking accountability, educating ourselves and our community, creating healing spaces, and engaging in advocacy (63). Evidence of the pain exists – now we must use its insights to move toward healing.

Bisexual+ women with chronic pain need support, resources, and healthcare that address both their mental distress and their chronic pain. Such supports and interventions must take an anti-sanist and anti-ableist approach that fundamentally trusts people in chronic pain about their experiences and views them as worthy regardless of their productivity. Within biomedicine, a crucial implication is that people with chronic pain should receive appropriate referrals and access to mental healthcare. These referrals should not dismiss the veracity of their chronic pain but rather acknowledge that chronic pain complexly both affects and is affected by mental health. As a step further, treatment protocols for complex chronic pain should combine biomedical, psychosocial, and holistic approaches in integrated care settings to avoid burdening patients with chasing referrals and navigating siloed health systems.

Another crucial implication of this study is that structural oppression and violence have significant roles in the creation of mental distress and chronic pain. While this link has been previously documented in quantitative literature, this study illustrates that the link between structural oppression and pain is also identified and *felt* by bisexual+ women themselves. Any true holistic treatment of chronic pain will involve structural work on the oppressive conditions that continue to disproportionately distress and pain marginalized people. Meaningful treatment of bisexual women’s chronic pain should involve systems-level work to dismantle biphobia, sexism, racism, ableism, and sanism.

Feminist disability studies scholars write that ambivalence, contradictions, and messiness are essential to understanding chronic illness and chronic pain. People in pain often want both medical treatment and social change (44, 64). Some people may believe that their mental health intimately affects their chronic pain, while others feel just as strongly that it does not. There is unlikely to be one perfect solution for all people with chronic pain. We need structural change in addition to tailored, holistic approaches that meet and believe each person individually.

## Data Availability

The datasets presented in this article are not readily available because they contain sensitive and potentially identifiable qualitative data. Requests to access the datasets should be directed to aster.a.harrison@gmail.com.

## Footnotes

1 Bisexual+ is an umbrella term that we use to encompass people with romantic and/or sexual attractions to people of more than one gender. These people might identify themselves as queer, pansexual, bisexual, or otherwise.

2 Biphobic discrimination describes unfair, unjust, or biased treatment on the basic of bisexual identity. Also known as binegativity, biphobia is a system of oppression which treats bisexuality as inferior to monosexual identities (homosexuality and heterosexuality).

3 The study’s definition of women included “cisgender and transgender women, as well as any others who identify as women.”

4 This interpretation of porosity of the bodies of people in pain is inspired by the work of Ahmed, Patsavas, and Siebers (32–34), discussed in greater depth in the discussion section.

5 In the 1980s, there were approximately 200 lesbian bars in the US. As of 2020, there were less than 20 (35). Gentrification, rising rents, and online dating have been cited as possible explanations for this phenomenon. The number of LGBTQ bars overall has declined in the past 20 years, but bars mostly or exclusively for women are particularly few, representing only 3.6 percent of LGBTQ bars (36).

## References

[1] Bostwick WB Harrison EA . Bisexual mental health. In: Rothblum ED , editor. Oxford Handbook of Sexual and Gender Minority Mental Health. Oxford, UK: Oxford University Press (2020).

[2] Chadwick AL Lisha NE Lubensky ME Dastur Z Lunn MR Obedin-Maliver J . Localized and widespread chronic pain in sexual and gender minority people—an analysis of the PRIDE study. Pain Med. (2024) 25:483–6. doi: 10.1093/pm/pnae023 38530776 PMC12104137

[3] Rikard SM Strahan AE Schmit KM Guy GP . Chronic pain among adults — United States, 2019–2021. MMWR Morb Mortal Wkly Rep. (2023) 72:379–85. doi: 10.15585/mmwr.mm7215a1 37053114 PMC10121254

[4] Tabaac AR Chwa C Sutter ME Missmer SA Boskey ER Austin SB . Prevalence of chronic pelvic pain by sexual orientation in a large cohort of young women in the United States. J Sex Med. (2022) 19:1012–23. doi: 10.1016/j.jsxm.2022.03.606 35508601 PMC9149035

[5] VanKim NA Flanders CE Bertone-Johnson ER . Sexual identity differences in chronic pain: Results from the 2019 to 2021 National Health Interview Survey. Am J Prev Med. (2024) 67:586–91. doi: 10.1016/j.amepre.2024.06.017 38908721 PMC11416321

[6] Zajacova A Grol-Prokopczyk H Liu H Reczek R Nahin RL . Chronic pain among U.S. sexual minority adults who identify as gay, lesbian, bisexual, or “something else. Pain. (2023) 164:1942–53. doi: 10.1097/j.pain.0000000000002891 37017364 PMC10436360

[7] Ross LE Salway T Tarasoff LA MacKay JM Hawkins BW Fehr CP . Prevalence of depression and anxiety among bisexual people compared to gay, lesbian, and heterosexual individuals: A systematic review and meta-analysis. J Sex Res. (2018) 55:435–56. doi: 10.1080/00224499.2017.1387755 29099625

[8] Salway T Ross LE Fehr CP Burley J Asadi S Hawkins B . A systematic review and meta-analysis of disparities in the prevalence of suicide ideation and attempt among bisexual populations. Arch Sex Behav. (2019) 48:89–111. doi: 10.1007/s10508-018-1150-6 29492768

[9] Taylor J Power J Smith E Rathbone M . Bisexual mental health and gender diversity: Findings from the “Who I Am” study. Aust J Gen Pract. (2020) 49:392–9. doi: 10.31128/ajgp-09-19-5073. Located at: informit.305562114289674. 32599996

[10] Flanders CE Wright M Khandpur S Kuhn S Anderson RE Robinson M . A quantitative intersectional exploration of sexual violence and mental health among Bi + people: Looking within and across race and gender. J Bisex. (2022) 22:485–512. doi: 10.1080/15299716.2022.2116515 37621766 PMC10449096

[11] Hooten WM . Chronic pain and mental health disorders. Mayo Clinic Proc. (2016) 91:955–70. doi: 10.1016/j.mayocp.2016.04.029 27344405

[12] Bondesson E Larrosa Pardo F Stigmar K Ringqvist Å Petersson IF Jöud A . Comorbidity between pain and mental illness – Evidence of a bidirectional relationship. Eur J Pain. (2018) 22:1304–11. doi: 10.1002/ejp.1218 29577509

[13] Kleykamp BA Ferguson MC McNicol E Bixho I Arnold LM Edwards RR . The prevalence of psychiatric and chronic pain comorbidities in fibromyalgia: An ACTTION systematic review. Semin Arthritis Rheumatism. (2021) 51:166–74. doi: 10.1016/j.semarthrit.2020.10.006 33383293

[14] Outcalt SD Kroenke K Krebs EE Chumbler NR Wu J Yu Z . Chronic pain and comorbid mental health conditions: Independent associations of posttraumatic stress disorder and depression with pain, disability, and quality of life. J Behav Med. (2015) 38:535–43. doi: 10.1007/s10865-015-9628-3 25786741

[15] Institute of Medicine (US) Committee on Lesbian, Gay, Bisexual, and Transgender Health Issues and Research Gaps and Opportunities . The Health of Lesbian, Gay, Bisexual, and Transgender People: Building a Foundation for Better Understanding. Washington (DC: National Academies Press (US (2011). [Internet]. (The National Academies Collection: Reports funded by National Institutes of Health). 22013611

[16] Breiding MJ Chen J Black M . Intimate partner violence in the United States – 2010. Atlanta, GA: National Center for Injury Prevention and Control, Centers for Disease Control and Prevention (2014) p. 1–96.

[17] Katz-Wise SL Mereish EH Woulfe J . Associations of bisexual-specific minority stress and health among cisgender and transgender adults with bisexual orientation. J Sex Res. (2017) 54:899–910. doi: 10.1080/00224499.2016.1236181 27834488 PMC6296471

[18] Feinstein BA Dyar C . Bisexuality, minority stress, and health. Curr Sex Health Rep. (2017) 9:42–9. doi: 10.1007/s11930-017-0096-3 28943815 PMC5603307

[19] Meyer IH . Prejudice, social stress, and mental health in lesbian, gay, and bisexual populations: Conceptual issues and research evidence. psychol Bull. (2003) 129:674–97. doi: 10.1037/0033-2909.129.5.674 12956539 PMC2072932

[20] Lick DJ Durso LE Johnson KL . Minority stress and physical health among sexual minorities. Perspect psychol Sci. (2013) 8:521–48. doi: 10.1177/1745691613497965 26173210

[21] Tong A Sainsbury P Craig J . Consolidated criteria for reporting qualitative research (COREQ): A 32-item checklist for interviews and focus groups. Int J For Qual Health Care. (2007) 19:349–57. doi: 10.1093/intqhc/mzm042 17872937

[22] Smith JA Flowers P Larkin M . Interpretative Phenomenological Analysis: Theory, Method and Research. Los Angeles: SAGE Publications Ltd (2009). p. 232.

[23] Harrison EA (2022). Experiences of bisexual+ women with chronic pain: an interpretive phenomenological analysis. University of Illinois Chicago. doi: 10.25417/uic.21516948.v1

[24] Otter.Ai. Available online at: otter.ai (Accessed September 1, 2021).

[25] Smith JA Flowers P Larkin M . Interpretative Phenomenological Analysis: Theory, Method and Research. London, UK: SAGE (2022). p. 240.

[26] American Psychological Association . Essentials of Interpretative Phenomenological Analysis (2021). Available online at: https://www.youtube.com/watch?v=5YM8Xxs8ucA (Accessed May 26, 2026).

[27] Montague J Holland F . Getting great data online workshop session 1: Introduction to Interpretive Phenomenological Analysis (IPA). (2020). (Accessed June 17, 2020).

[28] Atlas.ti Scientific Software Development GmbH . Atlas.Ti 8 for Windows. Available online at: https://atlasti.com/ (Accessed February 2, 2022).

[29] Vermont Psychiatric Survivors . What is Sanism? (2018). Available online at: https://www.facebook.com/VermontPsychiatricSurvivors/posts/what-is-sanismsanism-is-a-form-of-systemic-and-systematic-discrimination-and-opp/1890353791028954/ (Accessed February 1, 2022).

[30] Dusenbery M . Doing Harm: The Truth About How Bad Medicine and Lazy Science Leave Women Dismissed, Misdiagnosed, and Sick. 1st edition. New York, NY: HarperOne (2018). p. 400.

[31] Jackson G . Pain and Prejudice: How the Medical System Ignores Women—and What We can do About it. Vancouver, BC, Canada: Greystone Books Ltd (2019). p. 168.

[32] Ahmed S . The contingency of pain. Parallax. (2002) 8:17–34. doi: 10.1080/13534640110119597 37339054

[33] Patsavas A . Recovering a cripistemology of pain: Leaky bodies, connective tissue, and feeling discourse. J Literary Cult Disability Stud. (2014) 8:203–18. doi: 10.3828/jlcds.2014.16

[34] Siebers T . Disability Theory. Ann Arbor, MI, USA: University of Michigan Press (2008). p. 238.

[35] Berg A . After decades of declines, lesbian bars are having a renaissance. In: Nbc News (2023). Available online at: https://www.nbcnews.com/specials/lesbian-bars-resurgence-united-states/index.html (Accessed February 10, 2026).

[36] Mattson G . The changing mix of gay bar subtypes after COVID-19 restrictions in the United States, 2017 to 2023. Socius. (2023) 9:23780231231181902. doi: 10.1177/23780231231181902 37360679 PMC10280121

[37] Liegghio M . A denial of being: Psychiatrization as epistemic violence. In: LeFrançois BA Menzies RJ Reaume G , editors. Mad Matters: A Critical Reader in Canadian Mad Studies. Toronto, ON, Canada: Canadian Scholars’ Press Inc (2013). p. 122–9.

[38] Wanzo R . The Suffering Will Not be Televised: African American Women and Sentimental Political Storytelling. Albany, NY: SUNY Press (2009). p. 288.

[39] Werner A Malterud K . It is hard work behaving as a credible patient: Encounters between women with chronic pain and their doctors. Soc Sci Med. (2003) 57:1409–19. doi: 10.1016/S0277-9536(02)00520-8 12927471

[40] Dewar A . Chronic pain and mental illness: A double dilemma for all. J psy Nurs Ment Health Serv. (2007) 45:8–9. doi: 10.3928/02793695-20070701-01 17679308

[41] Raz M . The painless brain: Lobotomy, psychiatry, and the treatment of chronic pain and terminal illness. Perspect Biol Med. (2009) 52:555–65. doi: 10.1353/pbm.0.0121 19855124

[42] Lima DD Alves VLP Turato ER . The phenomenological-existential comprehension of chronic pain: Going beyond the standing healthcare models. Philos Ethics Humanit Med. (2014) 9:1–10. doi: 10.1186/1747-5341-9-2 24410937 PMC3996192

[43] Crow L . Including all our lives: Renewing the social model of disability. In: Morris J , editor. Encounters With Strangers: Feminism and Disability. London, UK: The Women’s Press (1996). p. 206–26.

[44] Wendell S . The Rejected Body: Feminist Philosophical Reflections on Disability. Hove, UK: Psychology Press (1996). p. 222.

[45] Price M . The bodymind problem and the possibilities of pain. Hypatia. (2015) 30:268–84. doi: 10.1111/hypa.12127 40046247

[46] Patsavas A . The logic of accounting: Pain, promises, prescriptions. Dissertation. Chicago, IL, USA: University of Illinois at Chicago (2017).

[47] Lewis TL Talila A. Lewis . Working Definition of Ableism (2021). Available online at: http://www.talilalewis.com/1/post/2021/01/january-2021-working-definition-of-ableism.html (Accessed February 2, 2022).

[48] Ross LE O’Gorman L MacLeod MA Bauer GR MacKay J Robinson M . Bisexuality, poverty and mental health: A mixed methods analysis. Soc Sci Med. (2016) 156:64–72. doi: 10.1016/j.socscimed.2016.03.009 27017092

[49] Harding S . Feminist standpoints. In: Handbook of Feminist Research: Theory and Praxis. SAGE Publications, Inc, 2455 Teller Road, Thousand Oaks California 91320 United States (2012). p. 46–64. Available online at: http://methods.sagepub.com/book/handbook-of-feminist-research/n3.xml (Accessed February 2, 2022). [Internet]. doi: 10.4135/9781483384740.n3

[50] Garland-Thomson R . Feminist disability studies. Signs: J Women Cult Soc. (2005) 30:1557–87. doi: 10.1086/423352 31878727

[51] Briggs S Cameron F . Psycho-emotional disablism, complex trauma, and women’s mental distress. In: Spandler H Anderson J Sapey B , editors. Madness, Distress, and the Politics of Disablement. Bristol, UK: Policy Press (2015). p. 115–26.

[52] Diamond S . What makes us a community? Reflections on building solidarity in anti-sanist praxis. In: LeFrançois BA Menzies RJ Reaume G , editors. Mad Matters: A Critical Reader in Canadian Mad Studies. Toronto, ON, Canada: Canadian Scholars’ Press Inc (2013). p. 64–77.

[53] Filson B . The haunting can end: Trauma-informed approaches in healing from abuse and adversity. In: Searching for a Rose Garden: Challenging Psychiatry, Fostering Mad Studies. Monmouth, UK: PCCS Books (2016). p. 20–4.

[54] Reeve D . Psycho-emotional disablism in the lives of people experiencing mental distress. In: Spandler H Anderson J Sapey B , editors. Madness, Distress, and the Politics of Disablement. Bristol, UK: Policy Press (2015). p. 99–112.

[55] Brown TT Partanen J Chuong L Villaverde V Griffin AC Mendelson A . Discrimination hurts: The effect of discrimination on the development of chronic pain. Soc Sci Med. (2018) 204:1–8. doi: 10.1016/j.socscimed.2018.03.015 29549869

[56] Dodge B Herbenick D Friedman MR Schick V Fu TC Bostwick W . Attitudes toward bisexual men and women among a nationally representative probability sample of adults in the United States. PloS One. (2016) 11:e0164430. doi: 10.1371/journal.pone.0164430 27783644 PMC5082634

[57] Dyar C Feinstein BA Zimmerman AR Newcomb ME Mustanski B Whitton SW . Dimensions of sexual orientation and rates of intimate partner violence among young sexual minority individuals assigned female at birth: The role of perceived partner jealousy. Psychol Viol. (2020) 10:411–21. doi: 10.1037/vio0000275 33163254 PMC7641336

[58] Dyar C Feinstein BA Anderson RE . An experimental investigation of victim blaming in sexual assault: The roles of victim sexual orientation, coercion type, and stereotypes about bisexual women. J Interpers Viol. (2021) 36:10793–816. doi: 10.1177/0886260519888209 31729280 PMC7225053

[59] Haviland MG Morton KR Oda K Fraser GE . Traumatic experiences, major life stressors, and self-reporting a physician-given fibromyalgia diagnosis. Psychiatry Res. (2010) 177:335–41. doi: 10.1016/j.psychres.2009.08.017 20382432 PMC2868959

[60] Sachs-Ericsson N Kendall-Tackett K Hernandez A . Childhood abuse, chronic pain, and depression in the National Comorbidity Survey. Child Abuse Negl. (2007) 31:531–47. doi: 10.1016/j.chiabu.2006.12.007 17537506

[61] Brennstuhl MJ Tarquinio C Montel S . Chronic pain and PTSD: Evolving views on their comorbidity. Perspect Psychiatr Care. (2015) 51:295–304. doi: 10.1111/ppc.12093 25420926

[62] Shirsat N Finney N Strutner S Rinehart J Higgins KE Shah S . Characterizing chronic pain and adverse childhood experiences in the lesbian, gay, bisexual, transgender, or queer community. Anesth Analgesia. (2024) 139:821–31. doi: 10.1213/ANE.0000000000006922 38412111

[63] Huxta A Center for Hunger Free Communities . A Look at Collective Trauma and Collective Healing (2021). Available online at: https://drexel.edu/hunger-free-center/news-events/voices-blog/2021/July/collective-trauma/ (Accessed February 1, 2022).

[64] Jones CE . The pain of endo existence: Toward a feminist disability studies reading of endometriosis. Hypatia. (2016) 31:554–71. doi: 10.1111/hypa.12248 40046247

